# Boosting Field Emission in Black Silicon via Gold Nanoparticle Decoration Guided by High-Fidelity 3D Morphological Simulation

**DOI:** 10.1021/acsami.5c13003

**Published:** 2025-10-30

**Authors:** Jia Li, Yuanpeng Zhang, Hui Wang, Zhengqin Zhao, Xinyi Liang, Dong Wang, Peter Schaaf, YongLiang Tang, Che Xu

**Affiliations:** † School of Physical Science and Technology, 56711 Southwest Jiaotong University, Chengdu 610031, China; ‡ Chair of Materials for Electrical Engineering and Electronics, Institute for Micro and Nanotechnologies MacroNano and Institute for Materials Science and Engineering, 26559 Technische Universität Ilmenau, 98693 Ilmenau, Germany

**Keywords:** nanomaterials, black silicon, field emission, 3D modeling, gold nanoparticles

## Abstract

Overcoming the inherent limitations of nanostructured black silicon (BS) for efficient field emission (FE) cathodes remains a significant challenge. Despite its promising nanostructured morphology with sharp, controllable tips amenable to mass production, BS suffers from a high work function (∼4.5 eV), poor electrical conductivity, and inadequate heat dissipation. These material properties lead to high threshold fields, unstable emission, and low current densities. Here, we demonstrate a simple and effective strategy to dramatically enhance the FE performance of BS through surface modification with gold nanoparticles (Au-NP). Critically, we overcome the complexity of simulating highly disordered BS emitters on nonplanar surfaces by developing a high-precision three-dimensional (3D) modeling approach based on parametrized scanning electron microscope (SEM) images. This model accurately captures the intricate surface morphology and enables the exploration of the electric field and current characteristics under varying Au-NP parameters. Guided by the simulations, we experimentally fabricated gold nanoparticle-decorated BS (Au-NP@BS) cathodes and achieved a significantly enhanced emission current density up to 4.01 mA/cm^2^. The close agreement between simulation predictions and experimental FE measurements validates this high-fidelity 3D modeling approach. This work not only provides a practical route to high-performance BS cathodes but also establishes a powerful computational tool for accurately mapping and optimizing the FE properties of complex, large-scale disordered nanostructures.

## Graphical abstract

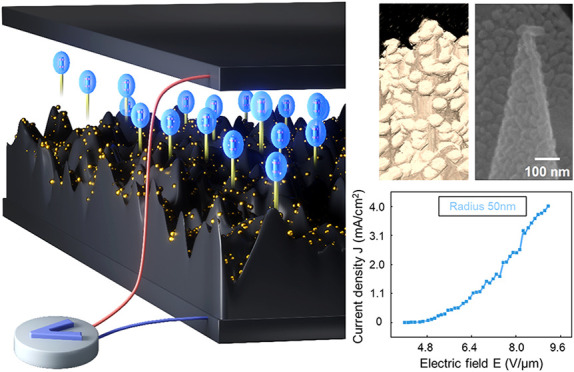

## Introduction

Black silicon (BS), a nanostructured silicon surface known for its unique topological characteristics, features a highly rough morphology with numerous sharp tips.^1^ Sometimes it is also called *Si nanograss*. Its excellent light-trapping abilities and nearly 100%^2^ broadband absorption from visible to near-infrared wavelengths make it highly valuable for applications in solar cells,^3,4^ photodetectors,^5^ photocatalysis,^6^ field-effect transistors,^7,8^ and sensing. Moreover, the abundant sharp nanotip structures on BS surfaces render it an ideal candidate for field emission (FE) cathodes^9–11^ in new electron sources, with its controllable geometry enhancing its application potential in electronic devices.^12,13^

However, due to the inherent material properties of black silicon, it suffers from an unstable emission current and low current density. To address these deficiencies, it has been optimized with surface modification and composite modification of this material to further enhance its performance.^14^ Nevertheless, selecting appropriate modification materials remains a critical issue in the current research. Ishikawa et al.^15^ conducted a systematic investigation into the FE properties of various metallic materials. By evaluating their maximum emission current, emission voltage, and surface conditions, they assessed the effective work function of these materials. It was found that gold emitters exhibit superior emission currents and stability under medium-voltage conditions. Based on this finding, gold-modified silicon nanostructured materials have garnered significant research attention in the field of field emission. Zhao et al.^16^ successfully fabricated Au–Si nanoparticles-decorated silicon nanowire arrays by depositing an Au thin film on silicon nanowire array substrates, achieving a low turn-on electric field of 1.95 V/μm. Chang et al.^17^ obtained well-aligned silicon nanocones using a one-step silver sputtering and dry etching process. After sputtering 10–20 nm thick Au and Pt films on the tips of the nanocones, the turn-on electric field decreased significantly from 4.2 to 2.9 MV/m, while the current density increased to 1.82 mA/cm^2^. However, theoretical studies on gold nanoparticle-decorated black silicon (Au-NP@BS) cathodes remain relatively scarce, particularly regarding the optimization of gold nanoparticle size, quantity, and their influence mechanisms on the field emission performance of BS. To enhance the field emission performance of BS cold cathodes, this study proposes combining a randomly distributed gold nanoparticle model with a realistic three-dimensional (3D) model of BS. Through simulation, the effects of the number, radius, and random distribution of gold nanoparticles on the field emission performance of BS cathode are systematically explored. The results guide the experimental fabrication of BS samples, and the modification-induced changes in their FE performance are tested experimentally to validate the accuracy of the 3D model and simulation results.

## Methods

### SEM Images, Static Statistical Analysis, and 3D Modeling of Black Silicon

First, to facilitate subsequent modeling and analysis, we conducted a quantitative characterization of the structural parameters of two black silicon (BS) samples based on the information from scanning electron microscope (SEM) images, including the characterized parameters cone height (*H*), top diameter (*R*), bottom diameter (*L*), and cone spacing (*d*), as shown in Figure 1a. Both sample 1 and sample 2 are fabricated using maskless reactive ion etching (RIE) approach with (100)-oriented n-type Si.

**Figure 1:**
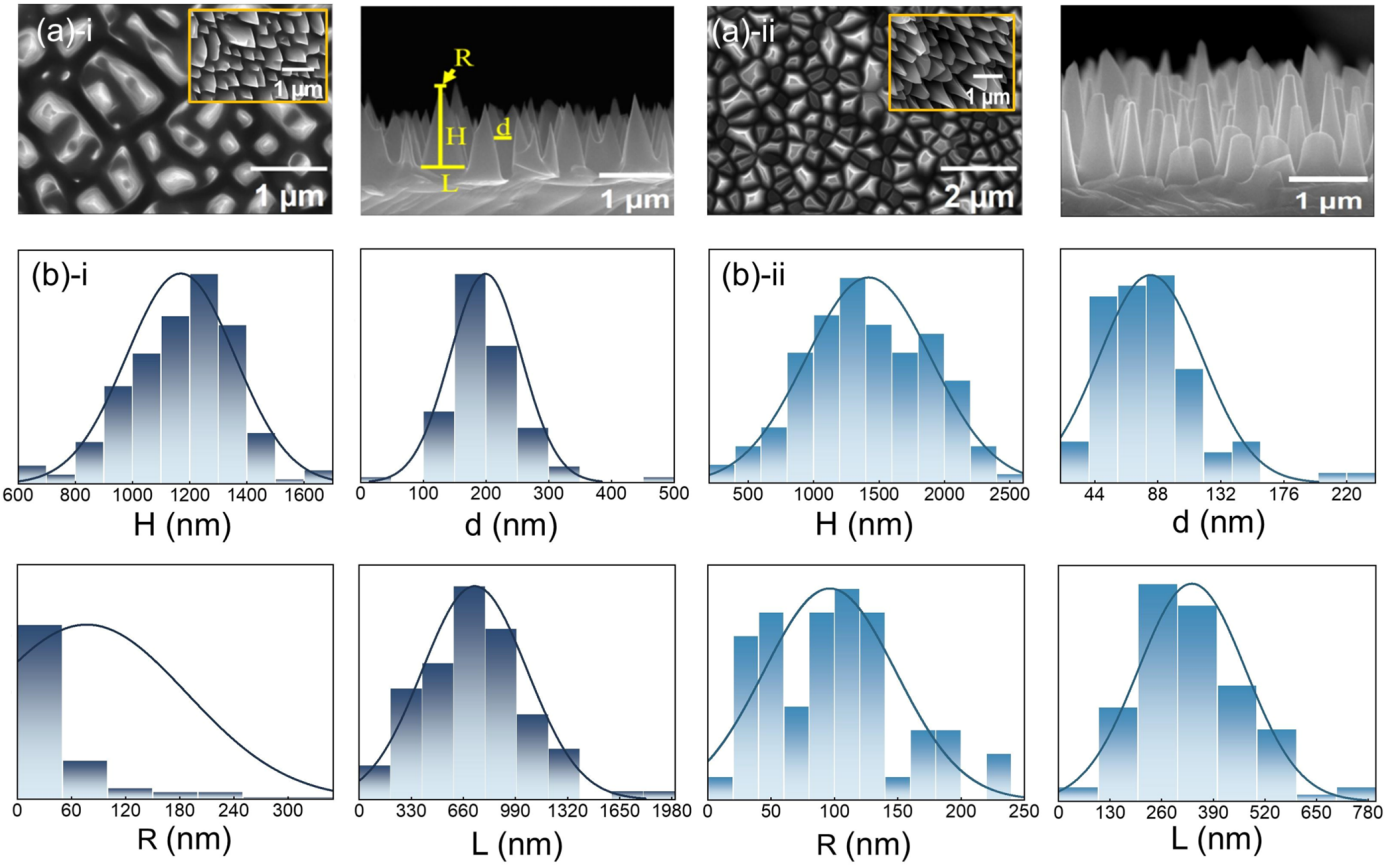
(a) From left to right: top view, 20° tilted-angle view (in the top right corner of the top view figure), and cross-sectional view. (b) Statistics of the cone height (*H*), top diameter (*R*), bottom diameter (*L*), and cone spacing (*d*). (i) Sample 1. (ii) Sample 2.

The depth differences between silicon cones are evaluated, and further the nanoscale geometric features of the cone profiles are extracted by analyzing the brightness distribution in top-view, cross-sectional, and 20° tilted SEM images (as shown in Figure 1a). Finally, the specific structural parameters of the two samples are obtained and a systematic statistical analysis are conducted. (The results are presented in Figure 1b and Table S1).

According to the statistical data, the cone heights of both Sample 1 and Sample 2 are within the range of 1000–2000 nm. The distribution of the top diameter (*R*) and bottom diameter (*L*) of Sample 1 is more uniform compared to that of Sample 2. Therefore, we extracted a uniform region with a cone height of (1100 ± 50) nm and consistent bottom diameters from the SEM image of Sample 1, and a nonuniform region with a cone height of (1400 ± 200) nm and larger variations in bottom diameters from the SEM image of Sample 2 for simulation and modeling. The extracted areas were both 2.7 μm × 2.7 μm, containing 27 effective cones.

Based on the top views of the extracted areas of the two samples and the geometric information obtained through statistical analysis, we successfully constructed uniform and nonuniform 3D models, as shown in Figure 2a. On this basis, by introducing the structural units of Au-NP, a 3D simulation model of Au-NP@BS was further generated. To verify the accuracy of this model, we carefully compared it with the top details of SEM images of Au-NP@BS sample prepared in the experiment, as shown in Figure 2b–d. The results indicate that this 3D model basically matches the observed microscopic morphological features of the top of the sample, which confirms its reliability for structural characterization. For more detailed information about the model construction, refer to Figure S1.

**Figure 2:**
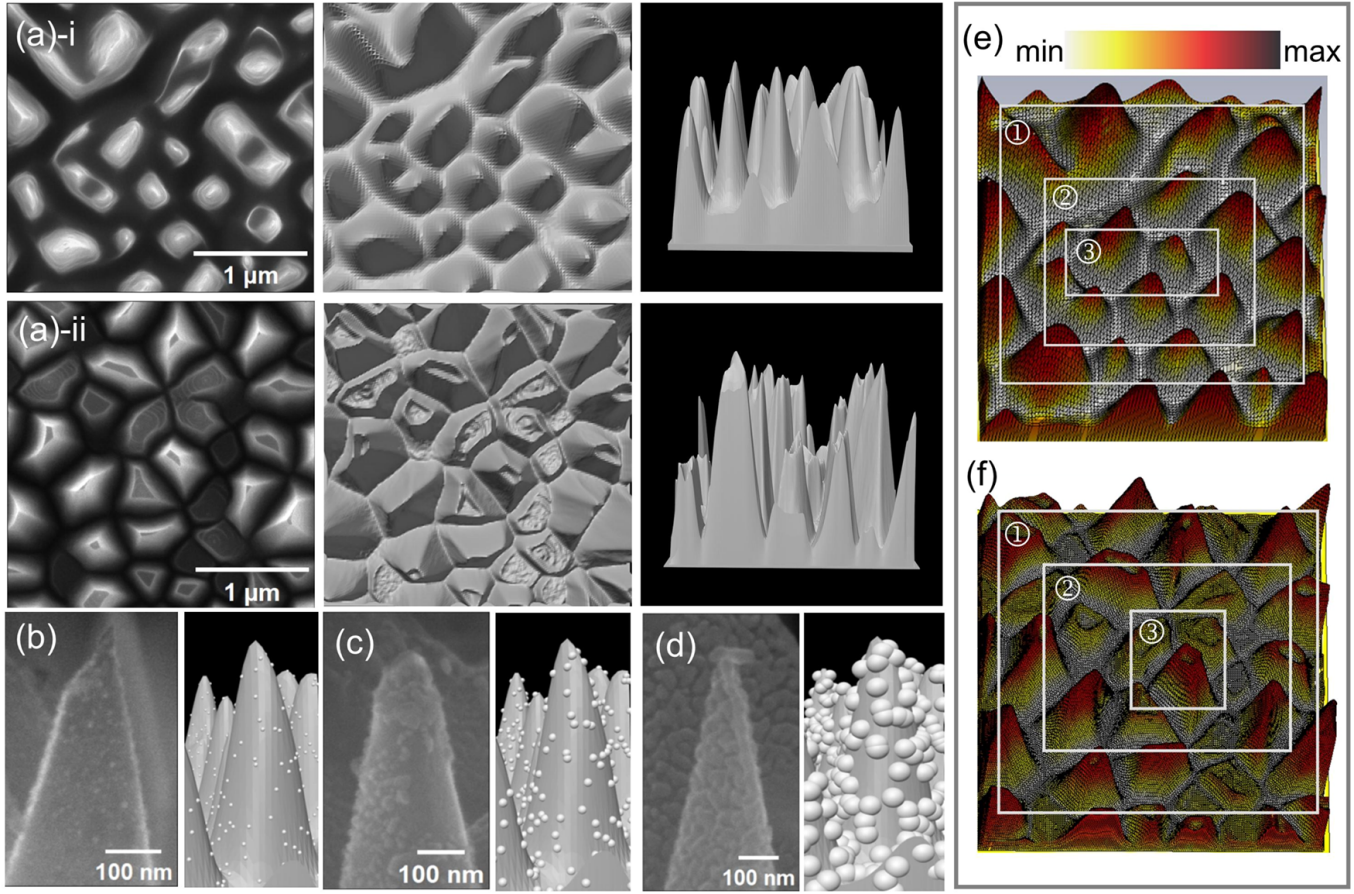
(a) From left to right: the top view of the extracted part in the SEM image, top view, and side view of the 3D model. (i) the uniform 3D model and (ii) the nonuniform 3D model. (b, c, d) SEM images of the top of the Au-NP@BS sample with different Au-NP radius and its corresponding 3D model. (e, f) Layered diagrams: (e) uniform model, (f) nonuniform model. 1: outer layer, 2: middle layer, 3: inner layer. The color bar intensity reflects the electric field strength.

## Results and Discussion

Based on the established model, numerical simulations were performed using an electromagnetic simulation method to investigate the FE characteristics of Au-NP@BS cathodes. By systematically varying the size and quantity of Au-NP, we investigated their influence mechanisms on the FE performance of BS. In the simulation, the material of the cone structure in the model was set as silicon (n-type silicon with a work function of 4.5 eV^18^), while the nanospheres were set as gold (with a work function of 5.1 eV^15^). The area of both the anode and cathode plates was 7 μm × 7 μm, and the interelectrode spacing was 7 μm. The radii of the Au-NPs were set to 10, 50, 100, and 200 nm, and their numbers were set to 100, 500, 1000, and 2000, respectively. These parameters were selected based on computational feasibility and distinct physical regimes. For Au-NP radius: 10–50 nm corresponds to small-to-medium-sized Au-NPs. Below 10 nm, visualization becomes unclear, and the enhancement effect approaches that of bare black silicon. At 100 nm, the Au-NPs are almost touching and cover the tip surface significantly. At 200 nm, Au-NPs coalesce to form a continuous metallic layer. For the number of Au-NPs: between 100 and 500 Au-NPs correspond to minimal to moderate coverage. At 1000 Au-NPs, nearly complete tip coverage is achieved, even for a particle size of 10 nm. When the number reaches 2000, a continuous metallic film forms regardless of the Au-NP size. To ensure consistency with subsequent experimental conditions, an external electrostatic field of 10 V/μm was applied in this typical vacuum diode structure.^19,20^

### Electric Field Simulation

Both models selected more than half of the surfaces on the tips of 27 cones, and the Au-NP distributed on these tips as the FE sites. The electric field data were extracted by using the ES processor. For the uniform model, the unmodified electric field was 1.69 × 10^2^ V/μm, while the electric field after modification with gold nanoparticles ranged from 1.69 × 10^2^ to 2.55 × 10^2^ V/μm. For the nonuniform model, the unmodified electric field was 1.43 × 10^3^ V/μm, while the electric field after modification with gold nanoparticles ranged from 1.43 × 10^3^ to 2.12 × 10^3^ V/μm. These results indicate that gold nanoparticles indeed enhance the electric field intensity of BS.^21–23^ To further investigate the underlying mechanism, both models were divided into three layers, as shown in Figure 2e,f.

Figure 3a,b, respectively, presents the statistical data of the electric field in the outer, middle, and inner layers of the two models, reflecting how the electric field varies with the radius and quantity of gold nanoparticles. The data trends on the left side of Figure 3 show that as the radius of the gold nanoparticles increases, the electric field at all tip positions decreases consistently and significantly in both models, regardless of variations in nanoparticle quantity. On the right side of Figure 3, when the radius exceeds 50 nm, increasing the quantity of nanoparticles further reduces the electric field across all locations, though the decrease is less pronounced at the tips. In summary, the variation in the radius of gold nanoparticles has a more pronounced effect on the electric field of BS.

**Figure 3:**
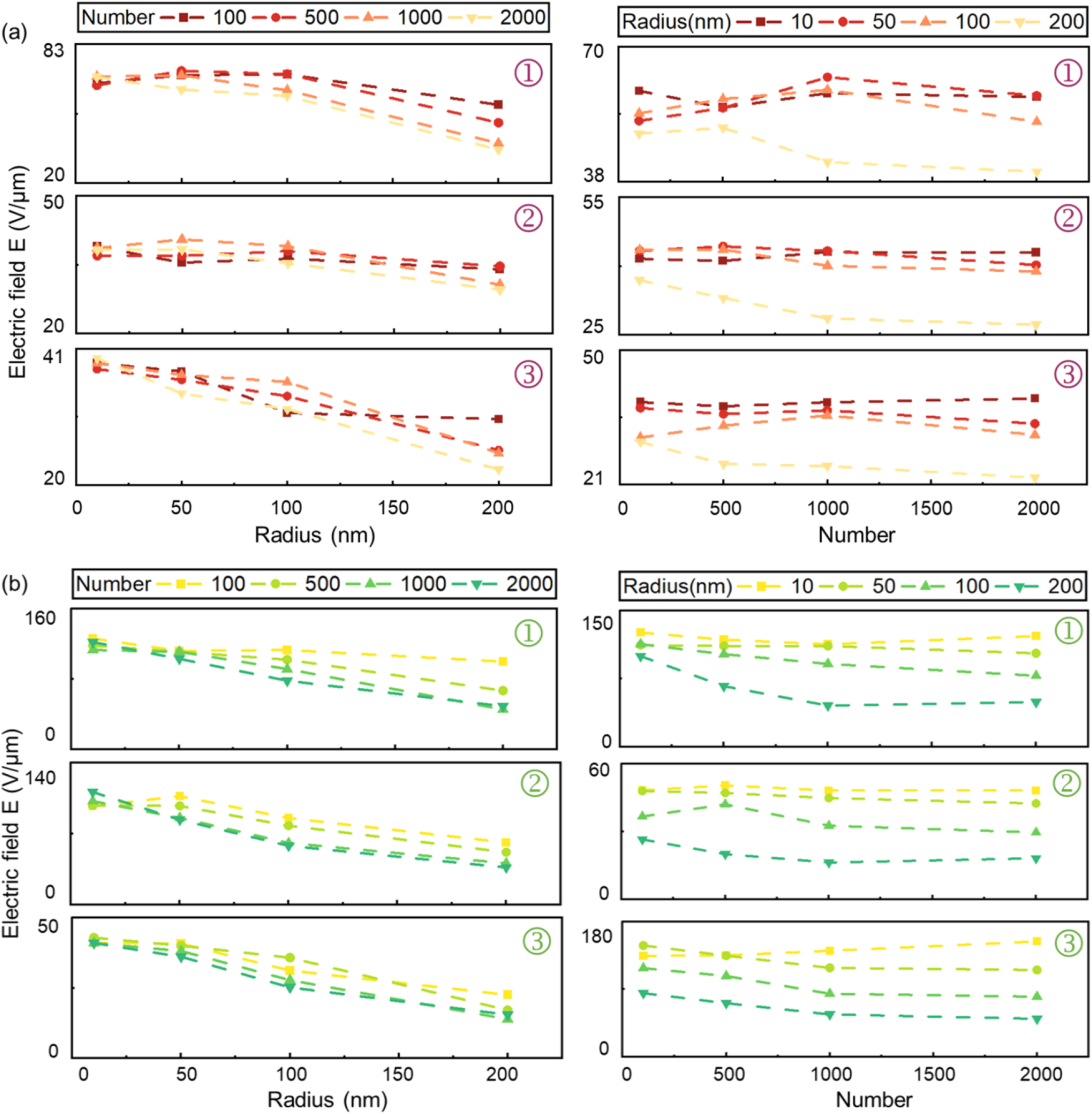
Influence of gold nanoparticle number and radius variations on electric fields in two BS models: (a) uniform model, (b) nonuniform model. 1: outer layer, 2: middle layer, and 3: inner layer.

As shown in Figure 4, the statistical data clearly reveal the correlation between the maximum electric field intensity at the tip of the cones and the radius of gold nanoparticles in BS arrays with varying numbers of gold nanoparticles. For the uniform model, it was found that regardless of the size of the gold nanoparticles, the position with the maximum electric field intensity at the cone tip almost always appears in the outer layer region. This phenomenon aligns with the edge effect,^24^ where the electric field intensity on the outer layer of the cone is generally higher than that on the inner layer.

**Figure 4:**
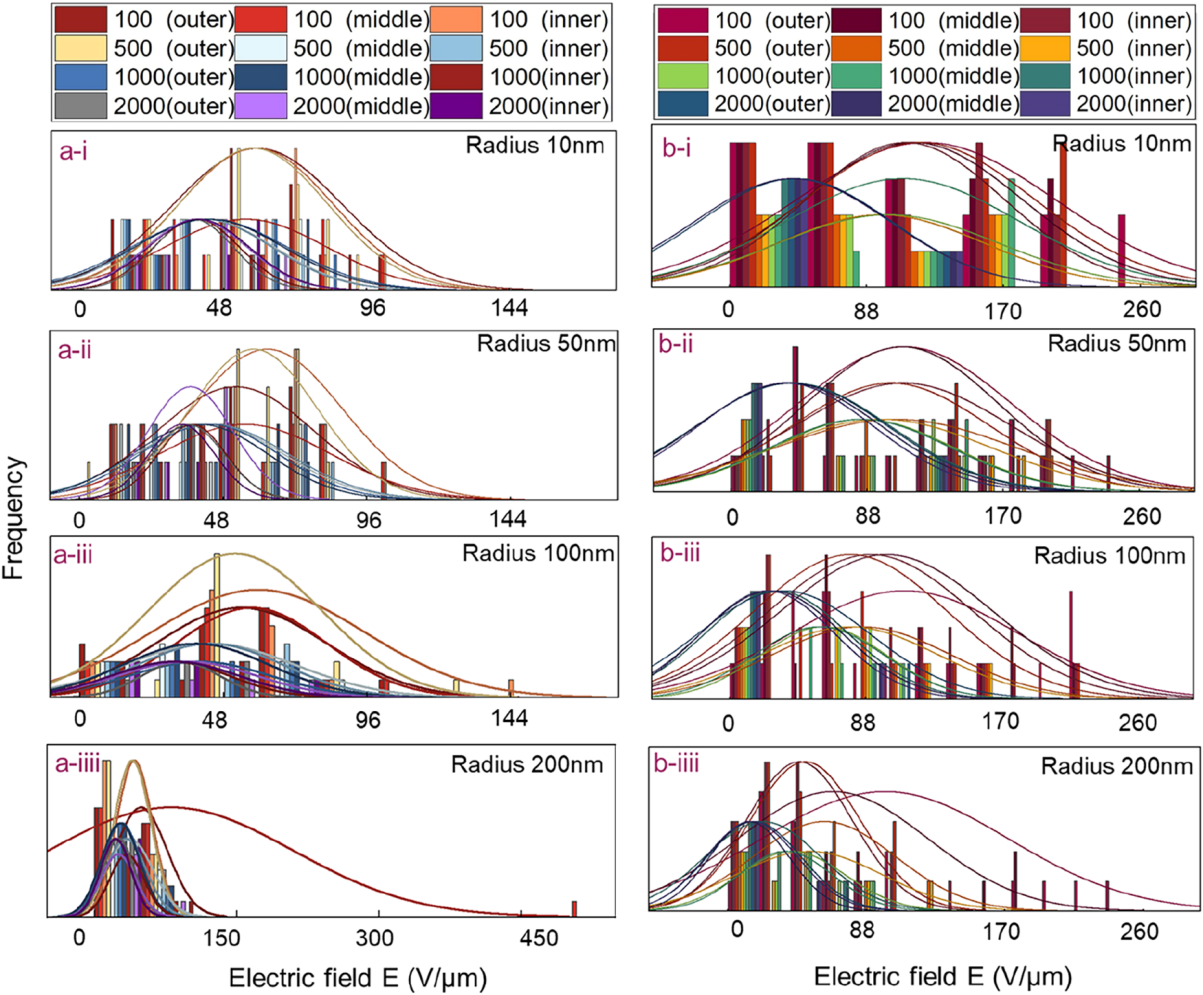
Variation of the electric field in each layer of the two BS models with the radius of gold nanoparticles: (a) uniform model, (b) nonuniform model. i: radius 10 nm, ii: radius 50 nm, iii: radius 100 nm, iiii: radius 200 nm.

Additionally, the data on the left side of the statistical graph indicate that as the radius of the gold nanoparticles increases, the frequency of occurrence of cones with lower electric field intensities also gradually rises. The reason for this is that when the radius of the gold nanoparticles is large, the nanoparticles almost completely cover the surface of the BS, thereby enhancing the shielding effect^25–27^ between the cones. These patterns also apply to the nonuniform model; however, it should be noted that as the particle radius increases, the distribution of low electric field intensities in the nonuniform model is not as concentrated as in the uniform model. This is because in the nonuniform model, there are significant differences in the heights of the cones (as shown in Figures S2–S5). Even when the gold nanoparticles almost entirely cover the tips, variations in the heights of the cones still exist, leading to a relatively weaker shielding effect.^28^ By comparing the statistical data of the two models, it can be observed that under moderate gold nanoparticle radius, the uniform model exhibits superior electric field enhancement effects.

### Current Simulation

Based on the conclusions derived from the electric field simulation, the radius of gold nanoparticles was set to 5, 10, 20, 50, 100, and 200 nm, while the number of gold nanoparticles was set to 100, 200, 500, and 1000, respectively. Over 200 points were selected as particle emission sources on the tips of each cone and on the distributed gold nanoparticles. All other simulation parameters remained consistent with those used in the electric field simulation. The current simulation was performed by using a PIC Solver simulator.

The calculated current density of the uniform model without modification is 1.54 mA/cm^2^, while that of the nonuniform model is 1.84 mA/cm^2^. As shown in Figure 5, the simulation results indicate that the current density in both models improves under the modification of the gold nanoparticles. When the number of gold nanoparticles is 1000 and the radius is 50 nm, the modification effect of both models reaches its optimum. At this point, the current density of the uniform model increases to 5.28 mA/cm^2^, and that of the nonuniform model increases to 5.50 mA/cm^2^. This result can be explained by the study of Saini et al.,^29^ which demonstrated that the incorporation of gold nanoparticles not only enhances the local electric field of BS array but also increases the number of emission sites at the tips. The prior electric field simulations in this study further confirm this point, following nanoparticle modification, both BS models exhibit improved electric fields: The uniform model shows an enhancement of 0–0.86 × 10^2^ V/μm, while the nonuniform model demonstrates an increase of 0–0.69 × 10^3^ V/μm. These findings align with previous research on the enhancement of electric fields through nanoparticle modification.^29,30^ During the FE process, BS samples emit electrons not only from their intrinsic tip structures but also from the gold nanoparticles deposited at the tips, which serve as additional emission sites. The combined effect of these contributions leads to a significant increase in the FE current density.

**Figure 5:**
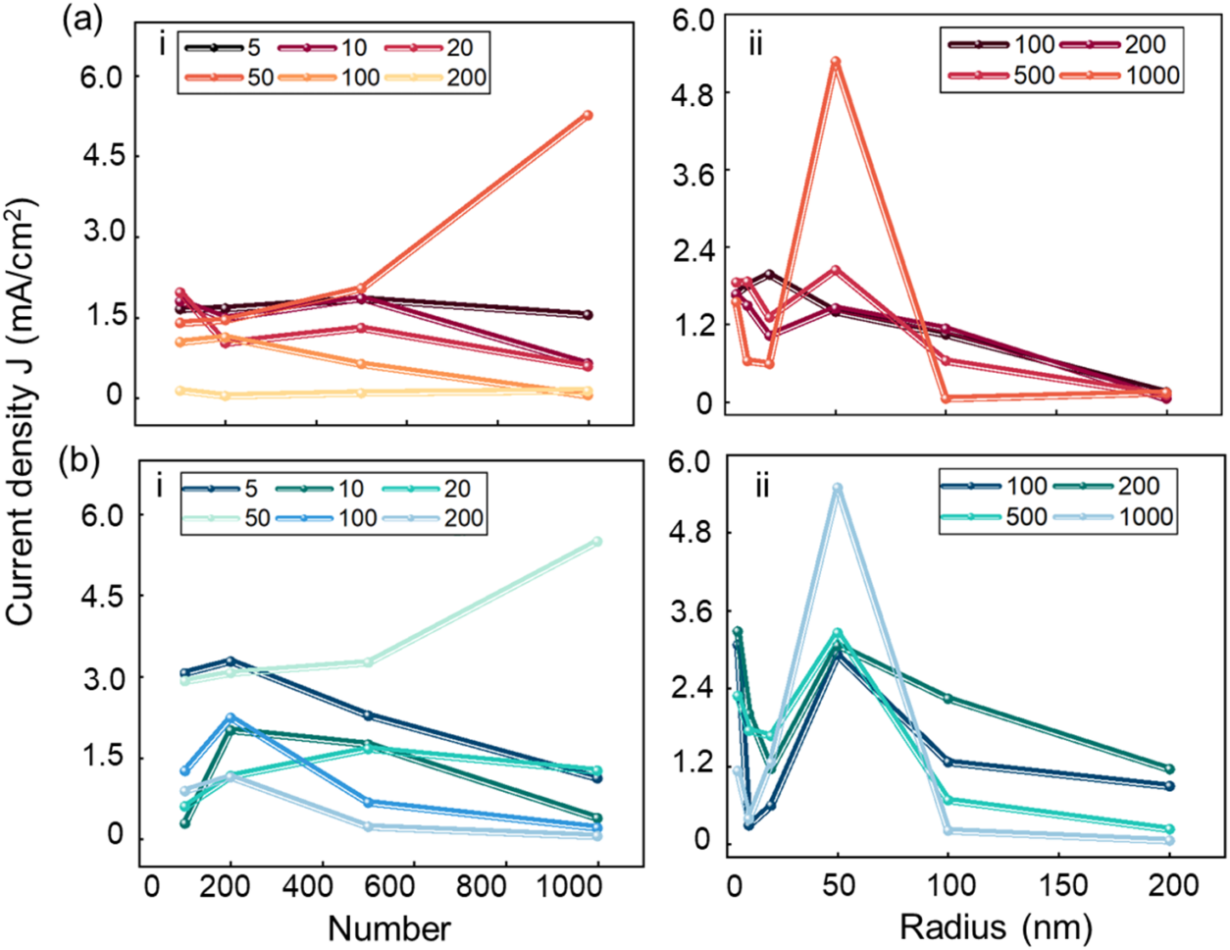
Variation of current with the number and radius of the gold nanoparticles: (a) uniform model, (b) nonuniform model. i: Number variation, ii: radius variation.

According to the statistical data presented in Figure 5, for both models, as the number of gold nanoparticles increases, the current density variation is significant for 50 nm gold nanoparticles but minimal for other radii. With an increasing number of nanoparticles, when the radius exceeds 50 nm, the current of both models decreases regardless of the quantity, with the nonuniform model showing a more pronounced trend. Regarding the final current density modulation effect, for 50 nm Au-NP at 1000-particle density, the uniform and nonuniform models show comparable current density increases (3.74 vs 3.66 mA/cm^2^), which indicates that the discrepancy between the two models is relatively minor.

### Experimental Preparation of Gold Nanoparticle-Decorated Black Silicon Samples and FE Experimental Test

The simulation results indicate that the radius of gold nanoparticles exerts a more significant effect on the field emission of BS. With the modification of gold particles, the uniform model demonstrates a superior field emission performance. However, comparisons of the simulation data from both models reveal negligible differences between them. Therefore, in the subsequent experimental preparation process, we did not consider the differences among BS samples but only set the radius of gold nanoparticles as the research variable.

Subsequently, we fabricated test samples as follows. First, BS with a disordered conical structure was prepared on an n-type 4 in. silicon wafer (P-doped, resistivity of 1–10 Ω·cm) using self-masked reactive ion etching (RIE) with SF_6_/O_2_ plasma chemistry. During the etching process, passivation material (SiO_*x*_F_*y*_) was deposited on the silicon surface and formed masking points, thereby inducing the disordered nanostructure of BS. This process does not require any lithography steps and is characterized by simplicity and high repeatability. Subsequently, gold nanoparticles with equivalent film thickness (on flat substrate) of 5, 20, and 50 nm were deposited on the silicon wafer surface via electron beam evaporation. Usually, nanoparticles can be formed on the nanostructured surface, and even a closed film cannot be obtained even with an equivalent film thickness of 50 nm. The preparation process is illustrated in Figure 6a.

**Figure 6:**
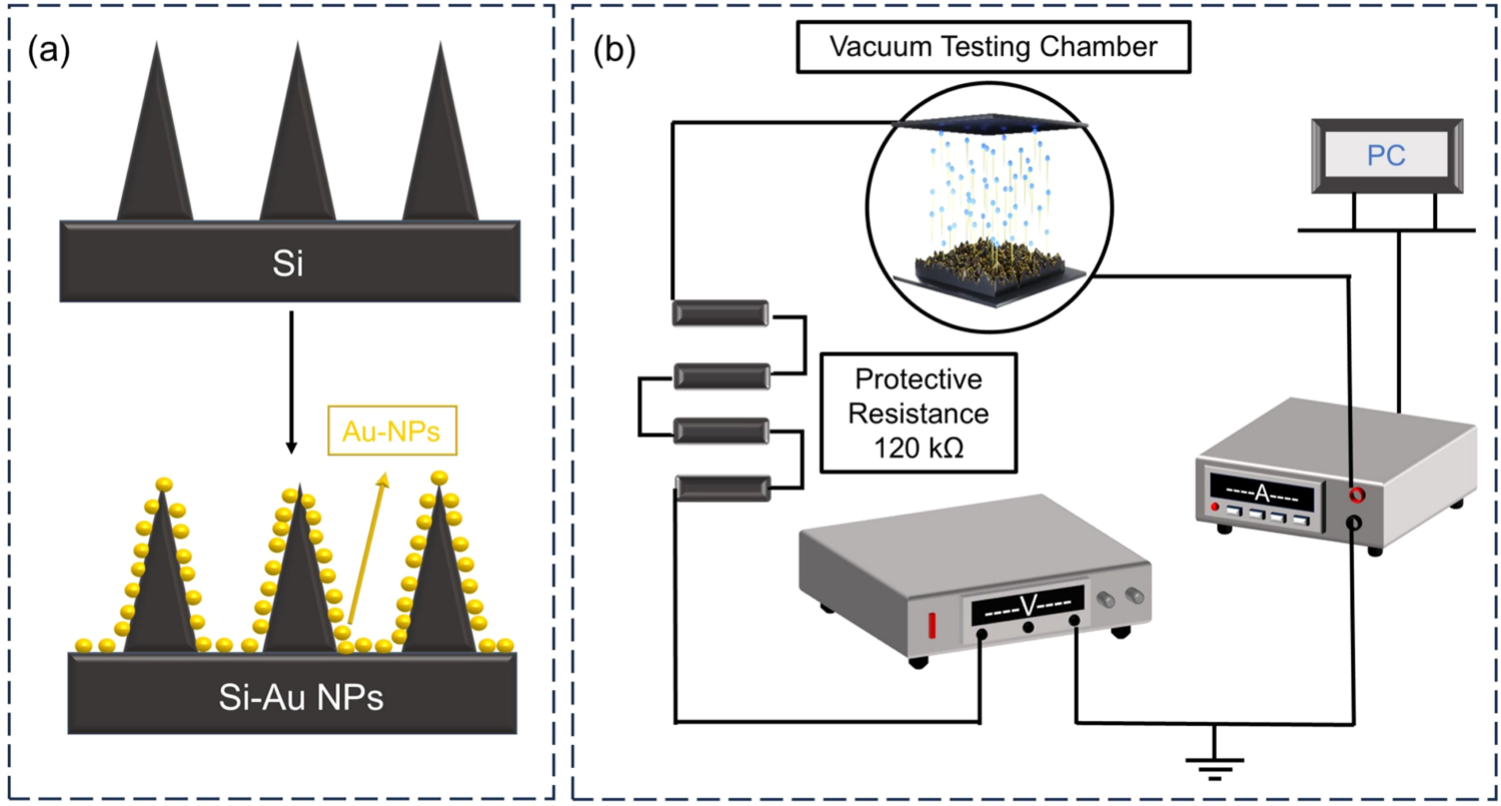
(a) Schematic diagram of the BS sample preparation process. (b) Circuit diagram for FE testing.

FE experiments for the Au-NP@BS cold cathode were conducted on a self-developed experimental platform. The cathode and anode of the experimental platform consisted of two sufficiently large rectangular stainless-steel plates separated by a 500 μm PTFE spacer. A groove with an area of 1 cm^2^ was located at the center of the cathode, and the test Au-NP@BS sample was fixed in the cathode groove using vacuum tape, with its surface aligned with the cathode plate. During the FE measurements, the device was placed in a vacuum chamber, which was connected in series with a high-voltage source and a picoamperemeter. The picoamperemeter automatically recorded the FE current as a function of the potential difference between the anode and cathode (*I*–*V*) with a recording interval of 0.5 s. A high voltage was applied to the anode in series with a 120 kΩ resistor to protect the picoamperemeter from damage caused by an electrical discharge. The vacuum level during testing was 8.4 × 10^–4^ Pa, and the experimental test circuit is shown in Figure 6b.

It can be observed from the *J*–*E* characteristic curve (as shown in Figure 7) that, compared with the unmodified black silicon (whose current density is only 1–2 mA/cm^2^), the modification with gold nanoparticles significantly enhances the FE performance of BS. Among them, the gold nanoparticles with a radius of 50 nm perform particularly prominently, and the corresponding current density can reach 4.01 mA/cm^2^. This experimental result is in good agreement with the conclusion of the simulation analysis. Meanwhile, we performed the current stability test on the Au-NP@BS sample modified with a Au-NP of 50 nm radius. As shown in Figure S6, under an electric field intensity of 5.08 V/μm, the current remained good stability during the nearly 2 h test with the current fluctuation within 7.04%.

**Figure 7:**
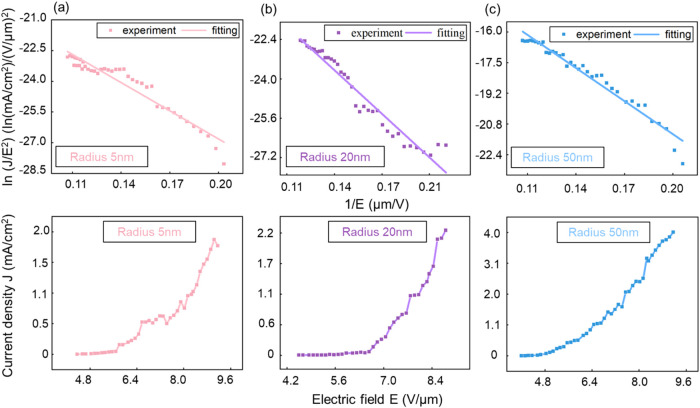
F–N theory and *J*–*E* characteristic curves of Au-NP@BS samples with different radii: (a) 5 nm, (b) 20 nm, (c) 50 nm.

The Fowler-Nordheim (F–N) theory^31^ is employed to describe the FE current when electrons tunnel through the barrier. For the FE process of N-type BS cathodes, the cathode emission current conforms to the F–N equation.^32^

1 $$J=\frac{AF^{2}}{\varphi }\exp\left(-\frac{B\varphi^{3/2}}{F}\right)$$

where *A* is assigned a value of 1.54 × 10^–6^ (A·V^–2^·eV) and *B* is assigned a value of 6.83 × 10^7^ (V·cm^–1^·eV^–3/2^),^33^ the field enhancement factor β is introduced, and the relationship between the local electric field *F* and the externally applied macroscopic electric field *E* can be expressed as

2 F=βE

by performing a mathematical transformation on the F–N equation and applying logarithmic processing to both sides, the following equation can be obtained

3 $$\ln\left(\frac{J}{E^{2}}\right)=\ln\left(\frac{A\beta^{2}}{\varphi }\right)-\frac{B\varphi^{3/2}}{\beta E}$$

based on the measured curve, after performing a mathematical transformation of the F–N formula, the field enhancement factor β can be obtained from the linear slope *K*. The slope *K* can be expressed as

4 $$K=-\frac{B\varphi^{3/2}}{\beta }$$

Among them, the effective work function φ of the Au-NP@BS sample was experimentally determined to be 3.92 eV. The field enhancement factor β of the Au-NP@BS sample with gold nanoparticles of 50 nm was calculated to be 4684.

## Conclusions

Black silicon with surface nanostructures loaded with Au nanoparticles (Au-NP@BS) was prepared and investigated as nano cold cathodes for field emission (FE). Precise 3D models were extracted from the samples, and the simulation results reveal the mechanism by which gold nanoparticle modification enhances the FE performance of BS. This study provides a powerful tool for revealing the structure–activity relationship between the micro-nanostructures of large-area, disordered “cone” arrays and FE performance. Based on this modeling approach, we simulated the FE characteristics of two BS models under varying gold nanoparticle radius and quantity, thoroughly investigating the mechanism by which gold nanoparticles enhance the FE performance of BS. Combining the simulation results, we fabricated BS samples using reactive ion etching (RIE) and electron beam evaporation techniques, followed by experimental testing of their FE properties. The experimental test results were in good agreement with the simulation data. Especially, the field emission current density of Au-NP@BS is successfully enhanced to 4.01 mA/cm^2^, nearly four times higher than that of undecorated BS, significantly enhancing the FE performance of BS. This outcome fully validates the consistency, rationality, and effectiveness of the proposed three-dimensional modeling method in both theoretical prediction and practical application.

## Supplementary Material



## Notes

Statistical results of the geometric structure information for the two types of black silicon (BS) samples (including cone density, height, top and bottom diameters of the cones, and cone spacing); detailed modeling process of the 3D models of BS and gold nanoparticles (Au-NP); analysis of the differences in the tips of the two types of BS models under the modification of gold nanoparticles with larger radius; and current stability test of Au-NP@BS cathode (PDF)
