# Supplementary material for: Boosting Field Emission in Black Silicon via Gold Nanoparticle Decoration Guided by High-Fidelity 3D Morphological Simulation
Source: ACS Appl Mater Interfaces. 2025 Oct 30;17(45):62420–8. doi: 10.1021/acsami.5c13003 (PMC12616592; doi:10.1021/acsami.5c13003)
Supplement: Supplementary file 1 [file am5c13003_si_001.pdf]

Supporting Information

Boosting Field Emission in Black Silicon via Gold Nanoparticle Decoration Guided by High-Fidelity 3D Morphological Simulation

Jia Li [a], *Yuanpeng Zhang* [a], *Hui Wang* [*, a], *Zhengqin Zhao* [a], *Xinyi Liang* [a],

Dong Wang [*, b], *Peter Schaaf* [b], *YongLiang Tang* [a], *Che Xu* [a]

[a] School of Physical Science and Technology, Southwest Jiaotong University, Chengdu 610031, China

[b] Chair of Materials for Electrical Engineering and Electronics, Institute for Micro and Nanotechnologies MacroNano and Institute for Materials Science and Engineering, Technische Universität Ilmenau, 98693 Ilmenau, Germany

* Email: wanghui@swjtu.edu.cn; dong.wang@tu-ilmenau.de

Statistical results of the geometric structure information for the two types of black silicon (BS) samples (including cone density, height, top and bottom diameters of the cones, and cone spacing); detailed modeling process of the 3D models of BS and gold nanoparticles (Au-NP); analysis of the differences in the tips of the two types of BS models under the modification of gold nanoparticles with larger radius; and current stability test of Au-NP@BS cathode.

1. Geometric Structure Information

Table S1 summarizes the statistical data on the geometric structure characteristics of two distinct N-type BS samples, including cone density, height, top and bottom diameters of cones, and inter-cone spacing. (1) These statistics allow for a comprehensive analysis of the geometric differences between the two BS samples. (2) Furthermore, this information serves as a critical foundation for the subsequent development of the two models.

Table S1. Specific structural parameters of two black silicon (BS) samples

Sample	Height H （nm）	Top diameters R （nm）	Bottom diameters L （nm）	Spacing D （nm）	Tip Density （$\times 10^8$/cm^2）
1	1300±200	20±10	325±100	175±50	3.9
2	1420±500	75±50	300±200	83±30	4.2

2. Detailed Process of 3D Modeling

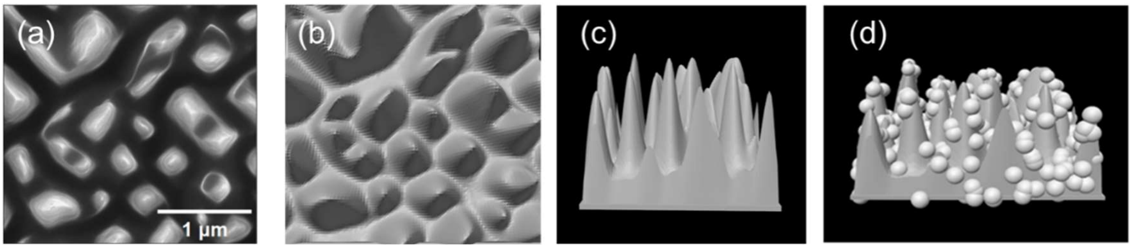

Figure S1. Modeling process: (a) SEM image of the scanned BS surface; (b) Computational model constructed based on statistical data of BS; (c) Finalized physical model after structural encapsulation; (d) Completed model incorporating gold nanoparticles.

Figure S1 shows the construction process of 3D modeling. The specific modeling steps are as follows:

(a). First, a three-dimensional animation drawing software was used to create planes with the same aspect ratio based on the extracted SEM images of BS nanomaterials. The number of segments in these planes was then adjusted accordingly.

(b). Next, the "replace" function in the software was utilized to read the grayscale information of the image and convert it into height information on the plane.

(c). Subsequently, the heights of the cones in the two models were adjusted according to the statistical information of the SEM cross-sections of the two models, and the models were encapsulated into solid models.

(d). Then, gold nanoparticles with target radius and quantity were randomly generated on the two models. The number of segments was adjusted to ensure smooth surfaces of the gold nanoparticles, and the models were exported as solid models.

(e). Finally, all points in the two models were optimized to eliminate irregularities. The models were subsequently imported into the corresponding electromagnetic simulation software for field emission simulation.

3. Two black silicon models with gold nano-particle radii of 100 nm and 200 nm

Figure S2, S3, S4, and S5 show the 3D models of gold nano-particles with radius of 100 nm and 200 nm and quantity of 500, 1000, and 2000, respectively. When the radius of the gold nano-particles is larger and their quantity is higher, the black silicon tips in both models are completely covered by the gold nano-particles, which reduces the distance between the tips and enhances the field screening effect. In comparison to the nearly uniform height array in the uniform model, the non-uniform model exhibits significant height variations due to its inherent structural differences. Even after being covered by gold nanoparticles, the height of the array in the non-uniform model remains uneven, resulting in a relatively weaker field screening effect.

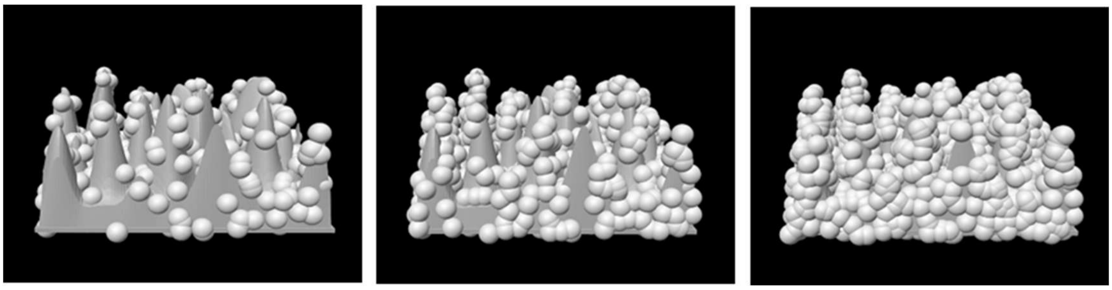

Figure S2. A front view of the uniform model with gold nanoparticles of 100 nm radius and quantities of 500, 1000, and 2000, respectively.

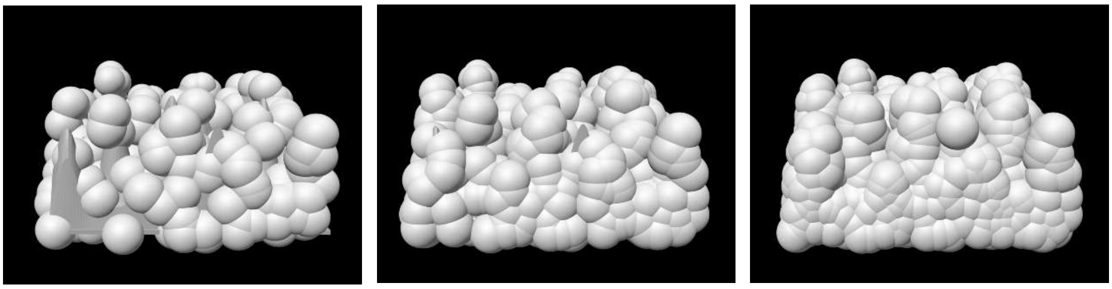

Figure S3. A front view of the uniform model with gold nanoparticles of 200 nm radius and quantities of 500, 1000, and 2000, respectively.

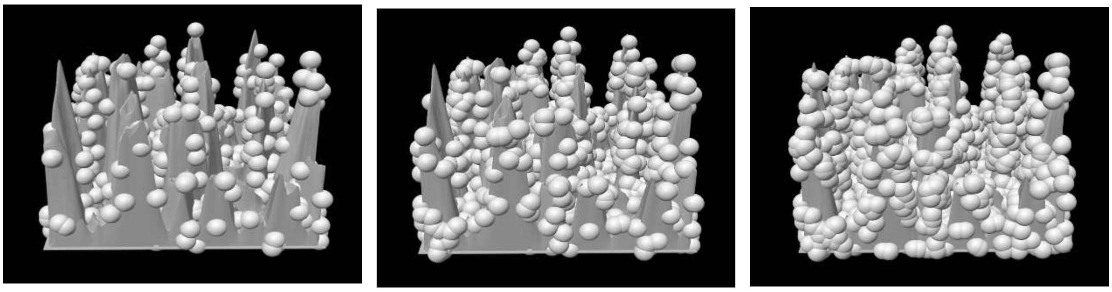

Figure S4. A front view of the non-uniform model with gold nanoparticles of 100 nm radius and quantities of 500, 1000, and 2000, respectively.

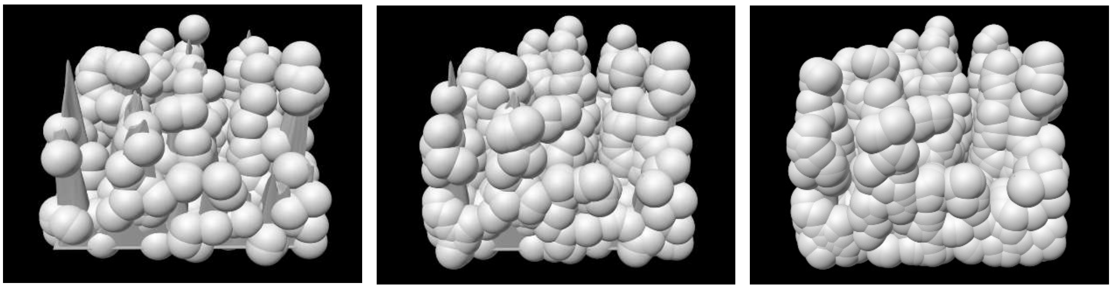

Figure S5. A front view of the non-uniform model with gold nanoparticles of 200 nm radius and quantities of 500, 1000, and 2000, respectively.

4. Current Stability Test

As shown in Figure S6, under an electric field intensity of 5.08 V/μm, the current of Au-NP@BS cathode modified with Au-NP of 50 nm radius remained good stability during the nearly two-hours test with the current fluctuation within 7.04%.

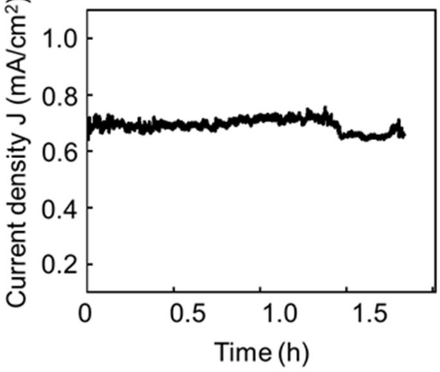

Figure S6. Current stability test of Au-NP@BS cathode.
